# Conditional Expression of E2A-HLF Induces B-Cell Precursor Death and Myeloproliferative-Like Disease in Knock-In Mice

**DOI:** 10.1371/journal.pone.0143216

**Published:** 2015-11-20

**Authors:** Jesús Duque-Afonso, Kevin S. Smith, Michael L. Cleary

**Affiliations:** Department of Pathology, Stanford University School of Medicine, Stanford, California, United States of America; B.C. Cancer Agency, CANADA

## Abstract

Chromosomal translocations are driver mutations of human cancers, particularly leukemias. They define disease subtypes and are used as prognostic markers, for minimal residual disease monitoring and therapeutic targets. Due to their low incidence, several translocations and their biological consequences remain poorly characterized. To address this, we engineered mouse strains that conditionally express *E2A-HLF*, a fusion oncogene from the translocation t(17;19) associated with 1% of pediatric B-cell precursor ALL. Conditional oncogene activation and expression were directed to the B-cell compartment by the Cre driver promoters *CD19* or *Mb1* (Igα, CD79a), or to the hematopoietic stem cell compartment by the *Mx1* promoter. *E2A-HLF* expression in B-cell progenitors induced hyposplenia and lymphopenia, whereas expression in hematopoietic stem/progenitor cells was embryonic lethal. Increased cell death was detected in *E2A-HLF* expressing cells, suggesting the need for cooperating genetic events that suppress cell death for B-cell oncogenic transformation. *E2A-HLF/Mb1*.*Cre* aged mice developed a fatal myeloproliferative-like disorder with low frequency characterized by leukocytosis, anemia, hepatosplenomegaly and organ-infiltration by mature myelocytes. In conclusion, we have developed conditional *E2A-HLF* knock-in mice, which provide an experimental platform to study cooperating genetic events and further elucidate translational biology in cross-species comparative studies.

## Introduction

Acute lymphoblastic leukemia (ALL) is a heterogenous disease comprised of several genetic subtypes, which are defined by genomic alterations including chromosomal aberrations, copy number variations and somatic mutations [1]. Genomic alterations confer the malignant clone different functional properties and are associated with prognosis, treatment response and relapse [2]. Recurring chromosomal translocations were the first genetic alterations characterized at the molecular level and in transgenic mice, and are associated with disease initiation and progression of hematological malignancies [3,4]. Although ALL is the most common childhood cancer [5], several chromosomal translocations defining ALL subtypes remain poorly characterized due to their low frequency.

The translocation t(17;19) codes for the chimeric fusion protein E2A-HLF (TCF3-HLF) [6,7], present in approximately 1% of pediatric B-cell precursor ALLs [8] and is associated with very poor prognosis [9]. The *E2A* (*TCF3*) gene codes for two key transcription factors, E12 and E47, which play important roles during B-cell development [10,11]. *HLF* is a basic leucine zipper (bZIP) transcription factor containing a proline and acidic amino acid rich (PAR) domain [12], which forms homodimers and heterodimers with other PAR protein family members [13,14]. The chimeric E2A-HLF fusion protein contains the two transcriptional activation domains AD1 and AD2 from E2A and the bZIP DNA-binding domain of HLF [15–17]. It is postulated that *E2A-HLF* oncogenic properties are partly due to the aberrant activation of *HLF* target genes and disruption of expression of *E2A* target genes [18].

Despite extensive efforts, no animal models have been developed recapitulating faithfully the human disease induced by E2A-HLF [19–21]. Here we employed conditional knock-in *E2A-HLF* mouse models to characterize the effects of oncogene expression in the B-cell, myeloid and hematopoietic stem cell compartments.

## Materials and Methods

### Mice

A conditional *E2A-HLF* allele was engineered to recombine human *HLF* cDNA into the mouse *E2A* locus to create an *E2A-HLF* fusion gene. Using a previously reported *E2A* conditional knockout construct [22] as template, a PGK-neo cassette was positioned downstream of the *E2A* gene, followed by a loxP site, splice acceptor sequence, and human *HLF* 3’cDNA sequences linked by an IRES element with the EGFP coding region. The targeted *E2A* allele encodes wild type *E2A*, but Cre-mediated recombination repositions the downstream *HLF*-EGFP cassette in frame with the *E2A* gene inducing expression of an *E2A-HLF* chimeric fusion transcript and EGFP under control of the *E2A* promoter. Transgenic *CD19*.*Cre (*Jackson laboratory [23]), *Mx1*.*Cre* (Jackson laboratory [24]), and *Mb1*.*Cre* (provided by *Dr*. David Allman, University of Pennsylvania, Philadelphia, PA, USA [25]) mice were intercrossed to generate *E2A-HLF/CD19*.*Cre*, *E2A-HLF/Mx1*.*Cre* and *E2A-HLF/Mb1*.*Cre* mice, respectively, on a C57BL/6 background. The *Mx1*.*Cre* promoter was leaky as shown by embryonic lethality and detection of GFP+ cells in the *E2A-HLF/Mx1*.*Cre* fetal liver cells, thus polyIC was not necessary to induce Cre recombinase. Moribund mice were humanely euthanized by carbon dioxide exposure followed by cervical dislocation. Criteria used to euthanize the mice were determined by the presence of signs of illness including general lymphadenopathy, lethargy, weight loss and shivering. Embryo mortality was assessed morphological by light microscopy, tissue maceration and overall pallor, which may have underestimated the incidence of embryo mortality.

### Histology and cytology

Tissues were fixed in 10% formalin, embedded in paraffin, sectioned and stained with hematoxylin-eosin. Blood smears were fixed with 100% methanol and stained with May-Grünwald solution (Sigma, St. Louis, MO) and Giemsa (Sigma) solution.

### Human cell lines

Human leukemia cell lines HAL-01 and REH (obtained from DSMZ, Braunschweig, Germany) were cultured in RPMI 1640 medium supplemented with 10% FBS, 100 U/ml penicillin/streptomycin, and 0.29 mg/ml L-glutamine. HAL-01 cells were authenticated for E2A-HLF expression by western blot.

### Flow cytometry and fluorescence activated cell sorting (FACS)

Cells were flushed from large bones or dissected from spleen and lymph nodes, filtered, rid of red blood cells in 1x RBC lysis buffer (eBioscience, San Diego, CA, USA), and washed twice with PBS. Flow cytometry was performed in LSR (BD Biosciences, San Jose, CA) using FACS DIVA software (BD Biosciences) and FlowJo (Treestar, Ashland, OR) for analysis. Cells were sorted for cell surface markers using a FACS Aria (BD Biosciences). Antibodies used for flow cytometry analysis and FACS sorting are listed in S1 Table. Lineage negative (Lin-) cells were detected with a cocktail of antibodies including anti-CD3, CD4, CD8, Mac1, Gr1, NK1.1, and Ter119.

### Apoptosis assays

Apoptosis assays were performed according to the manufacturer’s protocol using the annexin V apoptosis detection kit (eBioscience) and annexin V-V450 (BD Horizon) and propidium iodide (eBioscience). For intracellular staining of cleaved caspase 3, cells were fixed with 1.5% formaldehyde for 10 minutes at room temperature, permeabilized with 100% ice-cold methanol for 20 minutes on ice, washed twice with staining buffer and incubated with antibodies.

### Bone marrow transplantation assays

Secondary transplantation of bone marrow cells (1x10^6^) from MPD-like mice was performed by retro-orbital injection after sub-lethal irradiation (4.5 Gy) of 8–12 week-old C57BL/6 mice.

### Colony-forming assays

FACS-sorted Lin-CD19+CD43+ wild type or transgenic progenitor B cells (GFP+) from *E2A-HLF/Mb1*.*Cre* mice were cultured under lymphoid conditions in methylcellulose medium (M3234, Stem Cell Technologies, Vancouver, Canada) supplemented with 10 ng/ml IL-7 (Miltenyi Biotec, Auburn, CA). Total bone marrow cells from *E2A-HLF/Mb1*.*Cre* with MPD-like disease were cultured under myeloid conditions in methylcellulose medium (M3231, Stem Cell technologies) supplemented with the following cytokines from Peprotech (Rocky Hill, NJ, USA): IL-3 (10 ng/ml), IL-6 (10 ng/ml), GM-CSF (10ng/ml) and SCF (100 ng/ml) as previously described [26].

### RT-qPCR

RNA was isolated using the RNeasy Mini Kit (Qiagen, Valencia, CA, USA) and cDNA was synthesized using Superscript reverse transcriptase III kit (Life Technologies, Carlsbad, CA, USA) following the manufacturers recommendations. Relative expression was quantified using an ABI 7900HT Thermocycler using SYBR Green Master Mix (Applied Biosystems, Carlsbad, CA) and an annealing temperature of 60°C. Primers used to amplify target genes are listed in S2 Table. All signals were quantified using the ΔCt method and normalized to ΔCt-values of the *Actb* gene expression levels.

### Western blot analysis

Proteins were isolated using a modified RIPA lysis buffer containing 50 mM Tris HCl, 1% NP-40, 1% natrium-deoxycholate, 150 mM NaCl, 1 mM EDTA, 1 mM PMSF, 1 mM Na_3_VO_4_ and 1x Protease inhibitor cocktail (Roche). Equal amounts of protein were electrophoresed through 4–12% Bis/Tris gels (Life Technologies), transferred to Hybond-P membranes (GE Healthcare Biosciences, Pittsburgh, PA, USA), and immunodetected with rat anti-E2A (clone# 826927, clone R&D Systems, Minneappolis, MN), or rabbit anti-GAPDH (Cat. # G9545, Sigma) antibodies. Bands were detected by chemiluminescence using ECL Plus Western Blotting Detection System and HyperFilm (GE Healthcare Biosciences). Quantification by densitometry was performed using Image J Software [27].

### Statistics

Statistical differences between two groups were analyzed by two-sided Mann Whitney U test assuming a non-parametric distribution. A p-value below 0.05 was considered statistically significant. Statistical differences from Kaplan-Meier curves were analyzed by log-rank (Mantel Cox) test. Statistical analysis and graphs were performed using Graphpad prism software (Graphpad Inc., La Jolla, CA).

### Study approval

All experiments on mice were performed with the approval of and in accordance with Stanford’s Administrative Panel on Laboratory Animal Care (APLAC, Protocol 9839).

## Results

### Conditional *E2A-HLF* expression in the murine hematopoietic system

**Fig 1:**
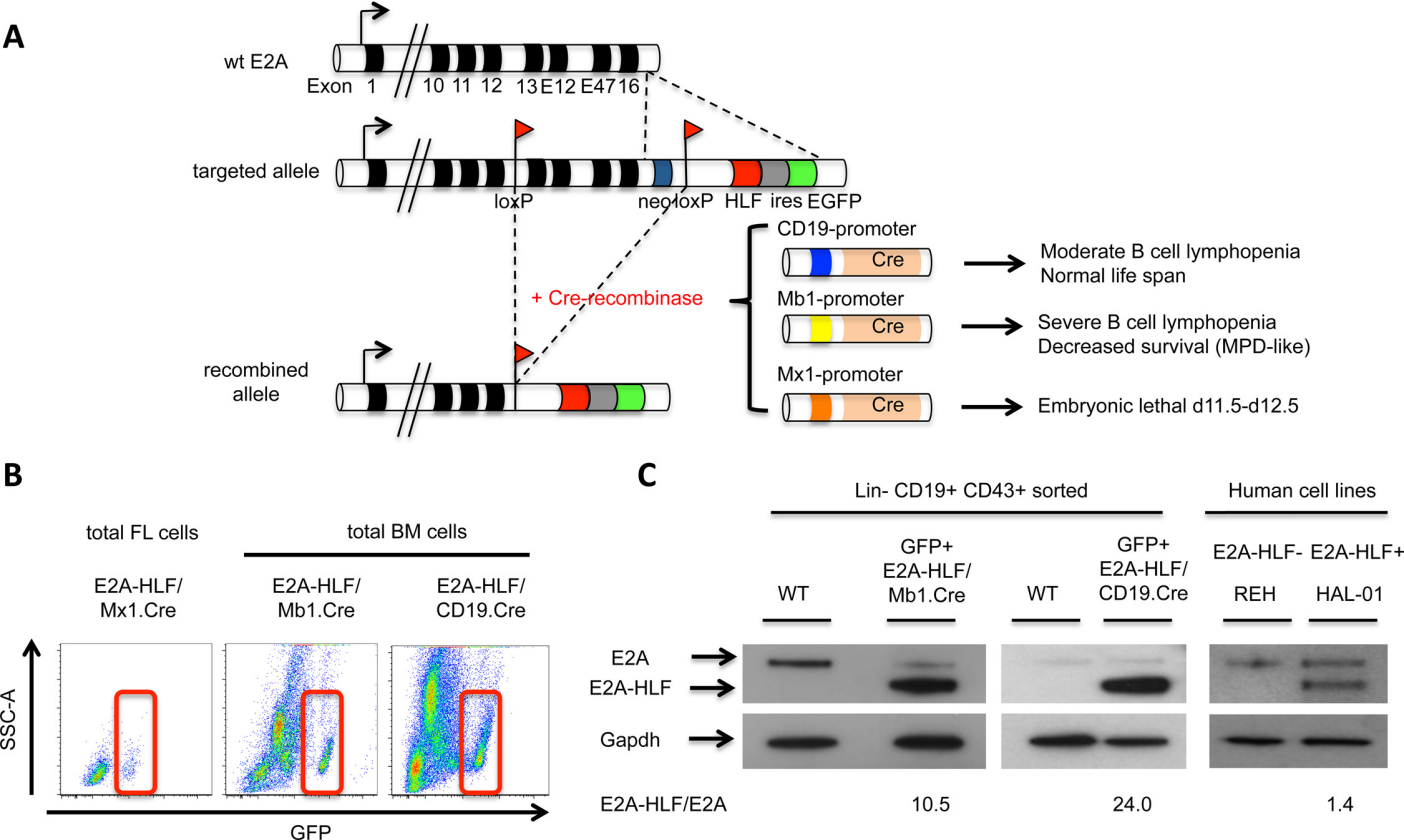
Conditional E2A-HLF transgenic mice. **(A)** Schematic representation of wild type, targeted, and recombined E2A alleles. After Cre recombinase expression, 3’ E2A exons (13, E12, E47 and 16) and the PGKneo cassette are deleted and the human HLF cDNA linked with EGFP by an IRES element is fused in frame to E2A. The expression of Cre-recombinase was driven by the B-cell specific promoters CD19 or Mb1 (CD79a, Igα), or in hematopoietic stem cells by the Mx1 promoter. The phenotypes arising depending on the Cre-recombinase driven promoter are indicated on the right. MPD-like, myeloproliferative disease like. **(B)** Flow cytometry analysis shows GFP expression from fetal liver (FL) cells of an E2A-HLF/Mx1.Cre embryo and from total bone marrow (BM) of E2A-HLF/Mb1.Cre and E2A-HLF/CD19.Cre mice. **(C)** Representative western blots show E2A and E2A-HLF protein levels in FACS-sorted progenitor B cells from wild-type (WT, lin-CD19+CD43+) and 2-month-old transgenic (lin-CD19+CD43+GFP+) E2A-HLF/Mb1.Cre and E2A-HLF/CD19.Cre mice. For comparison, E2A and E2A-HLF expression is shown from the non-E2A-HLF cell line REH and from the E2A-HLF+ cell line HAL-01. GAPDH was used as loading control. The E2A/E2A-HLF ratio (shown below) was determined by densitometry.

To study the molecular contributions of *E2A-HLF* in leukemogenesis, we developed mouse strains that conditionally activate and express the *E2A-HLF* chimeric fusion gene. Expression of the conditional *E2A-HLF* allele was achieved in B cell progenitors using Cre recombinase under the control of B-cell specific promoters *CD19* or *Mb1* (*Igα*, *CD79a*) or in hematopoietic stem/progenitor cells using the *Mx1* promoter (Fig 1A). To track *E2A-HLF* recombination and expression at the single-cell level, the *GFP* gene was positioned after an IRES element in the targeted allele. GFP+ cells were detected in bone marrow from *E2A-HLF/CD19*.*Cre* and *E2A-HLF/Mb1*.*Cre* transgenic mice and in fetal liver cells from *E2A-HLF/Mx1*.*Cre* embryos (Fig 1B). E2A-HLF protein expression was detected in sorted bone marrow B-cell progenitors from *E2A-HLF/CD19*.*Cre* and *E2A-HLF Mb1*.*Cre* 2-month old transgenic mice. Notably, the ratio E2A-HLF/E2A was 10–20 fold in knock-in mice compared to approximately 1.5 fold in the E2A-HLF+ human cell line HAL-01 (Fig 1C). In *E2A-HLF/Mb1-Cre* mice, GFP expression was detected in ~20% of mature B cells (CD19+B220+) and 85% of B cell progenitors (Lin-CD19+CD43+) compared to fewer than 1% of mature myeloid Mac1+ cells and T cell subsets of peripheral blood. However, ~20% of the immature myeloid cells (Gr1+Mac1-) of the bone marrow were GFP+ (S1 Fig). Thus, E2A-HLF was preferentially but not exclusively expressed in the B-cell lineage of knock-in mice.

### Conditional *E2A-HLF* mice develop B-cell lymphopenia and hyposplenia

**Fig 2:**
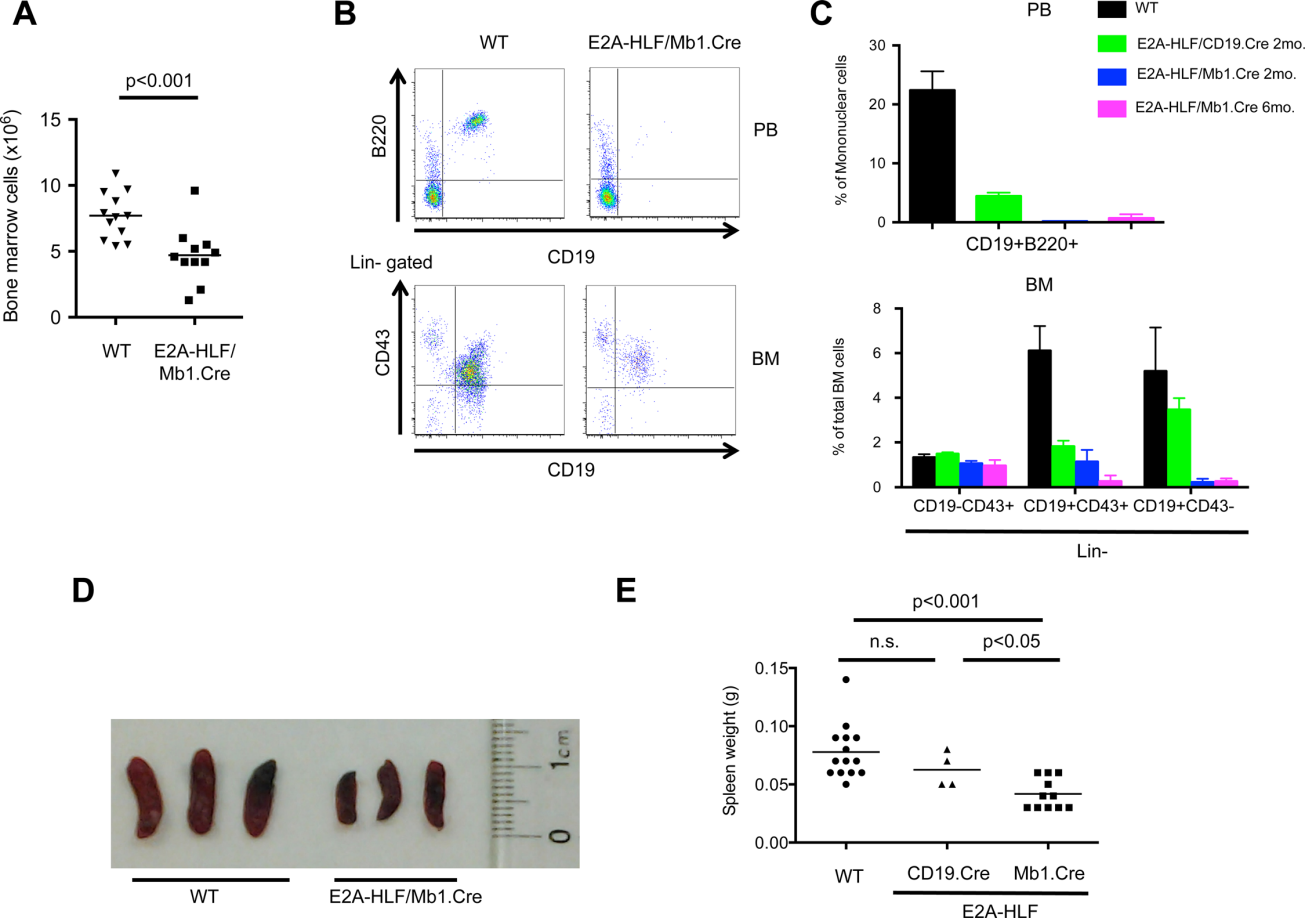
Conditional E2A-HLF transgenic mice develop B cell lymphopenia and hyposplenia. **(A)** Total bone marrow cells from 2-month-old wild-type (WT, n = 12) and E2A-HLF/Mb1.Cre (n = 11) transgenic mice were enumerated by trypan blue exclusion assay. Horizontal bars denote the mean. Statistical analysis was done by Mann-Whitney U test. **(B)** Dot plots from flow cytometry analysis show mature B cell (B220+CD19+) subpopulations in peripheral blood (PB, upper panel) and progenitor B cell subpopulations (Lin-CD19-CD43+, Lin-CD19+CD43+, Lin-CD19+CD43-) in bone marrow (BM, lower panel) of representative wild-type and E2A-HLF/Mb1.Cre transgenic mice. Lin, lineage markers (CD3, CD4, CD8, NK1-1, Mac1, Gr1, Ter119). **(C)** Graph summarizes relative frequencies of mature B cells in peripheral blood (upper panel) and progenitor B cells in bone marrow (lower panel) of wild-type (WT, n = 9), E2A-HLF/CD19.Cre 2-month-old mice (n = 4), E2A-HLF/Mb1.Cre 2-month-old (n = 6) and 6-month-old (n = 3) mice. Columns denote mean and bars denote standard error of the mean. **(D)** Spleens are shown for representative WT (n = 3) and transgenic E2A-HLF/Mb1.Cre mice (n = 3). **(E)** Graph shows spleen weights from WT (n = 14), E2A-HLF/CD19.Cre (n = 4) and E2A-HLF/Mb1.Cre (n = 11) mice, horizontal bars denote the mean. Statistical analysis was done by Mann-Whitney U test; n.s., not significant.

To assess the cellular effects of conditional *E2A-HLF* expression during lymphopoiesis, peripheral blood and bone marrow cells from knock-in mice at different ages were analyzed by flow cytometry. Conditional expression of *E2A-HLF* consistently reduced total bone marrow cell numbers, mature B cells numbers in the peripheral blood and B cell progenitors in the bone marrow (Fig 2A–2C). Hence, the spleen size was smaller and spleen weight was lower in *E2A-HLF* conditional mice compared to age-matched wild type controls (Fig 2D and 2E). Total spleen cells as well as mature B-cells and immature B-cell progenitors were reduced in *E2A-HLF/Mb1*.*Cre* transgenic mice (S2 Fig). Of note, B cell lymphopenia as well as hyposplenia were much more pronounced in two and six month-old *E2A-HLF/Mb1*.*Cre* mice compared to *E2A-HLF/CD19*.*Cre*, which are predicted to activate *E2A-HLF* expression at a later time point during B cell development. Thus, *E2A-HLF* causes B cell lymphopenia, although we cannot exclude that the differences observed in B cell progenitors are partly due to *E2A* haploinsufficiency following conditional activation of the *E2A-HLF* fusion allele.

### Conditional *E2A-HLF* mice show increased cell death in B cell progenitors

**Fig 3:**
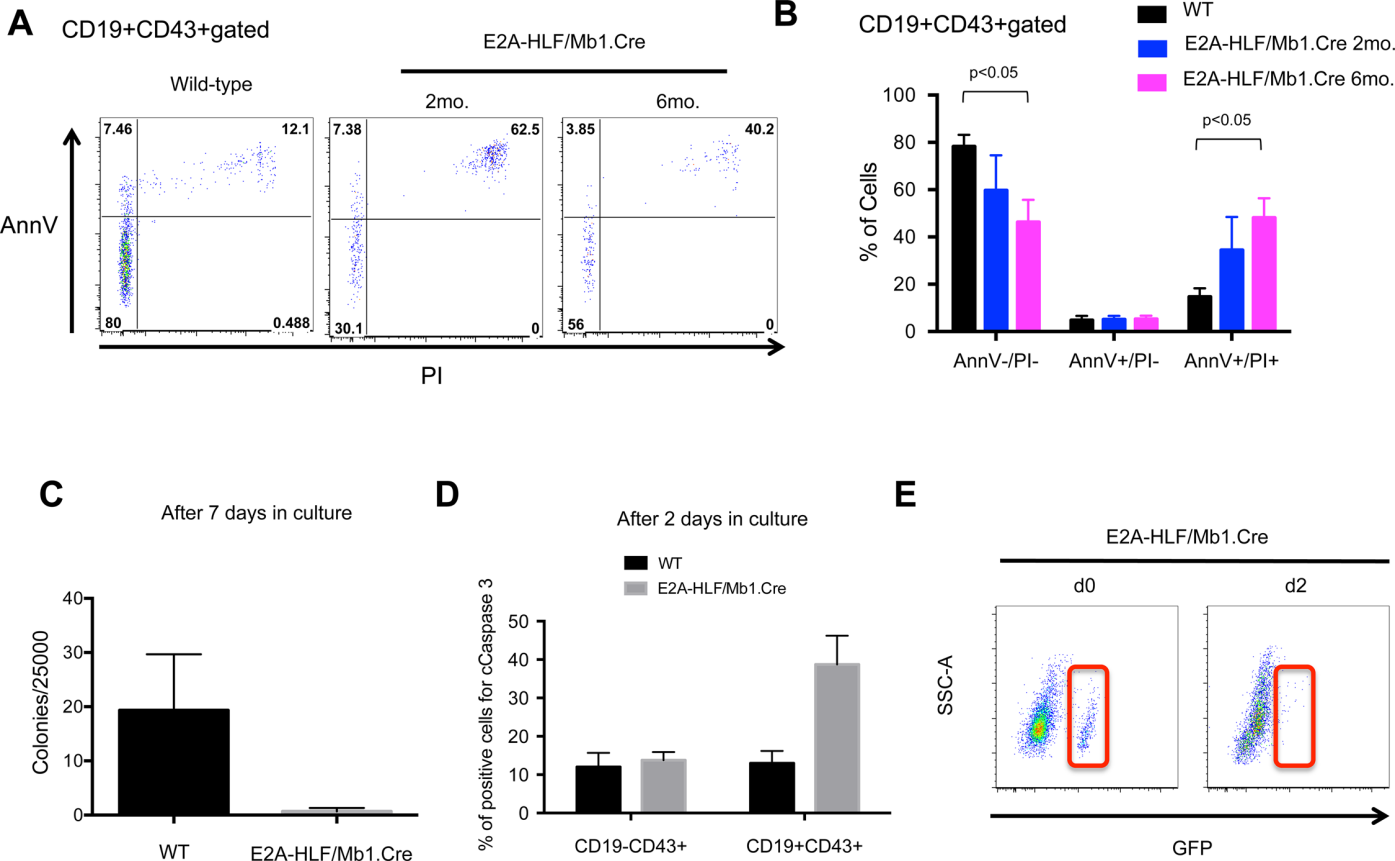
E2A-HLF conditional mice show increased B cell progenitor death. **(A)** Dot plots show annexin V (AnnV) and propidium iodide (PI) staining in CD19+CD43+ gated cells of representative wild type, E2A-HLF/Mb1.Cre 2-month-old and 6-month-old transgenic mice. **(B)** Graph summarizes relative live (Ann-/PI-), apoptotic (AnnV+/PI-) and necrotic (AnnV+/PI+) fractions in CD19+CD43+ gated cells of bone marrow samples from wild type (WT, n = 5), E2A-HLF/Mb1.Cre 2-month-old (n = 3) and 6-month-old (n = 3) mice. Columns denote mean and bars denote standard error of the mean. Statistical analysis was performed using Mann-Whitney U test. **(C)** Bone marrow cells were sorted from wild type (WT, n = 3), E2A-HLF/Mb1.Cre transgenic 2-month-old (n = 3) and cultured in methylcellulose enriched with IL-7 (10 ng/ml). Colonies were counted after 7 days. Columns denote mean and bars denote standard error of the mean. **(D)** Total bone marrow cells from WT (n = 3) and 2 month-old E2A-HLF/Mb1.Cre mice (n = 3) were cultured in methylcellulose enriched with IL-7 (10 ng/ml). After 2 days, cells were analyzed for cleaved caspase3 (cCaspase 3) in B cell progenitor subpopulations (CD19-CD43+ and CD19+CD43+). **(E)** Flow cytometry analysis shows GFP expression in bone marrow cells from E2A-HLF/Mb1.Cre transgenic mouse before and after 2 days of culture. A representative from three experiments is shown.

The decrease of mature and progenitor B cells and decreased spleen size suggested that *E2A-HLF* might induce apoptosis and cell death as previously described in transgenic *E2A-HLF* mouse models [19,20]. The fraction of apoptotic (annexin V+/PI-) and necrotic (annexinV+/PI+) cells in CD19+CD43+ B cell progenitors of wild type and *E2A-HLF/Mb1*.*Cre* bone marrow was analyzed by flow cytometry. Transgenic *E2A-HLF* mice showed a significant increase of necrotic cells, together with a decrease in live cells of the gated B cell progenitors (Fig 3A and 3B, S3 Fig). To address the proliferative capacity of B cell progenitors expressing E2A-HLF, lin-CD19+CD43+ cells were FACS-sorted from wild type and *E2A-HLF/Mb1*.*Cre* mice and cultured in methylcellulose containing IL-7. *E2A-HLF/Mb1*.*Cre* B-cell progenitors formed very few colonies compared to wild type (Fig 3C). After two days of cell culture, *E2A-HLF/Mb1*.*Cre* progenitor B cells showed a modest increase of the apoptotic marker cleaved caspase 3 in the CD19+CD43+ subpopulation (Fig 3D) and E2A-HLF expressing GFP+ cells decreased dramatically (Fig 3E). In summary, E2A-HLF induces cell death in B-cell progenitors of transgenic mice in vivo and in vitro.

### Conditional expression *E2A-HLF* during early hematopoiesis is embryonic lethal

**Fig 4:**
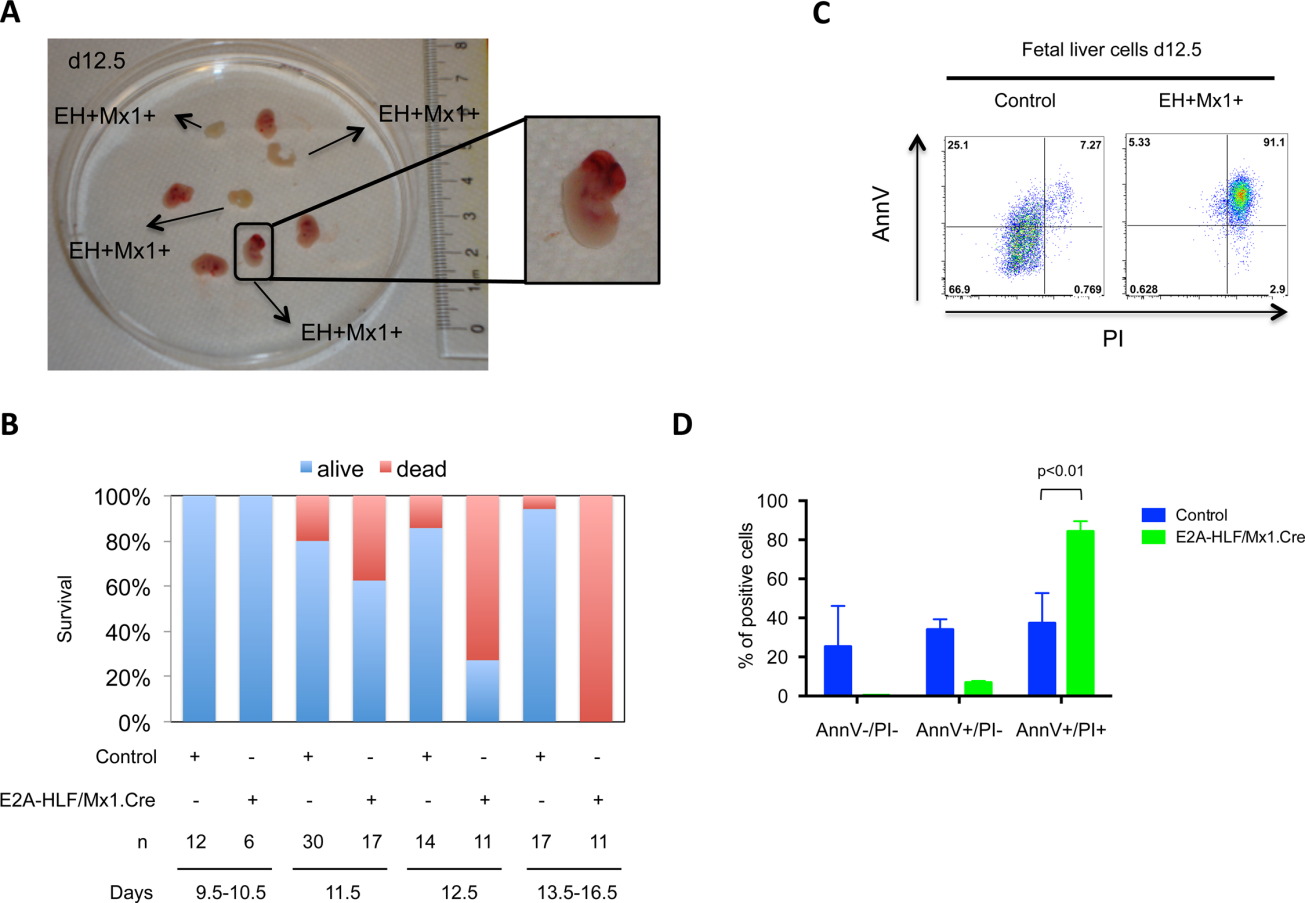
Expression of E2A-HLF in fetal liver hematopoietic progenitors results in embryonic lethality. **(A)** Image shows embryo morphology (n = 8) at E12.5 E2A-HLF/Mx1.Cre (EH+Mx1+) embryos. An E2A-HLF/Mx1.Cre embryo in the lower part of the picture shows cranial hemorrhage and three other E2A-HLF/Mx1.Cre embryos were not viable. **(B)** Graph summarizing mortality of embryos depending on their genotype and developmental stage. Controls comprise wild type, E2A-HLF+/Mx1.Cre- and E2A-HLF-/Mx1.Cre+ embryos. A total of 118 embryos were analyzed. **(C)** Fetal liver cells of control (n = 3) and E2A-HLF/Mx1.Cre (n = 3) embryos at E11.5 were stained with annexin V (AnnV) and propidium iodide (PI) for apoptosis analysis. A representative experiment is shown. **(D)** Graph summarizes annexin V/propidium staining from fetal liver cells. Columns denote mean and bars denote standard error of the mean. Statistical analysis was done by Mann Whitney U test.

To study the effects of *E2A-HLF* in hematopoietic stem cells, conditional *E2A-HLF* mice were crossed with *Mx1*.*Cre* mice, which activate Cre recombinase in this cellular compartment [28]. Matings yielded 83 pups, but none were *E2A-HLF/Mx1*.*Cre*, suggesting that expression of *E2A-HLF* in hematopoietic stem cells during embryonic development might be embryonic lethal. Timed pregnancies resulted in 118 embryos between E9.5 to E16.5 of embryonic development. All *E2A-HLF/Mx1*.*Cre* embryos were alive at E10.5 but their viability decreased between E11.5-E12.5. At E13.5, none of the *E2A-HLF/Mx1*.*Cre* embryos were viable (Fig 4A and 4B). Of note, some embryos showed cranial hemorrhage suggesting a defect in embryonic hematopoiesis. No other developmental abnormalities were observed macroscopically and histologically in *E2A-HLF/Mx1*.*Cre* mutant embryos at this stage of development.

We hypothesized that E2A-HLF induces cell death in embryonic hematopoietic stem cells similar to progenitor B cells from *E2A-HLF/CD19*.*Cre* and *E2A-HLF/Mb1*.*Cre* lines. To test this, fetal liver cells were harvested at E11.5 and analyzed for the fraction of apoptotic/necrotic cells by annexin V/propidium iodide (PI) staining (Fig 4C and 4D). Hematopoietic stem/progenitor cells were mainly necrotic (annexin V+/PI+) in *E2A-HLF/Mx1*.*Cre* embryos compared to control embryos from the same mating (mean 84% vs 37%, p-value<0.01). These data further suggest conditional E2A-HLF expression is toxic in hematopoietic stem/progenitor cells.

### *E2A-HLF* transgenic mice developed myeloproliferative-like disease with low penetrance

**Fig 5:**
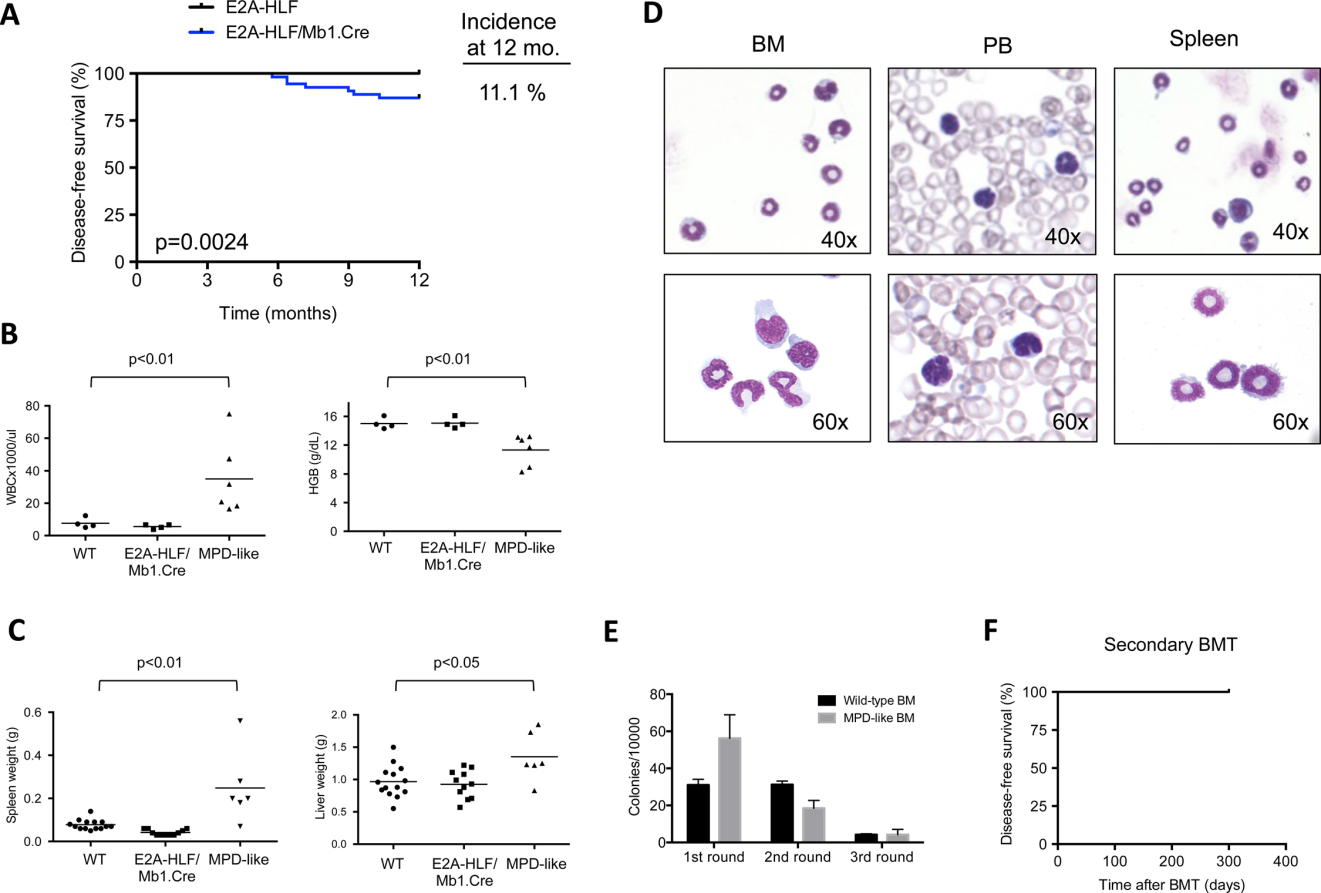
E2A-HLF/Mb1.Cre transgenic mice develop myeloproliferative-like disease. **(A)** Kaplan-Meier plots show disease-free survival of conditional E2A-HLF/Mb1.Cre mice (n = 54) and control E2A-HLF mice (n = 67). The incidence of myeloproliferative disease (MPD)-like at 12 months is shown on the right. Statistical difference between curves were calculated by log-rank (Mantel-Cox) test. **(B)** White blood cell counts (WBC, left panel) and hemoglobin concentration (HGB, right panel) at MPD-like disease presentation (n = 6) compared to wild type (n = 4) and healthy transgenic mice (n = 4). Each dot represents a mouse and horizontal bars denote the mean of analyzed mice. Statistical analysis was performed by Mann-Whitney U test. **(C)** Graph shows spleen (left panel) and liver weights (right panel) of wild type (WT, n = 9), healthy transgenic (n = 8) and MPD-like (n = 6) mice. Each dot represents a mouse and horizontal bars denote the mean of analyzed mice. Statistical analysis was performed by Mann-Whitney U test. **(D)** May-Grünwald Giemsa staining of cytospins from bone marrow (BM), spleen and peripheral blood smear (PB) show morphology of MPD-like cells. **(E)** Bone marrow cells were cultured in methylcellulose enriched with a myeloid cytokine-cocktail. Compact colonies were counted every seven days and then, cells were re-plated. Columns denote mean and bars standard error of the mean of triplicates. A representative of two bone marrow cells from WT and MPD-like mice is shown. **(F)** Secondary bone marrow transplantation (BMT) of 100,000 bone marrow cells from a mouse with MPD-like disease in five sublethally irradiated recipients. After 10 months of follow-up, no mice developed disease.

*E2A-HLF/Mb1*.*Cre* mice showed decreased disease-free survival (Fig 5A) due to myeloproliferative-like disease (MPD-like), which fulfilled the diagnosis criteria from the hematopathology subcommittee of Mouse Models of Human Cancers Consortium [29]. MPD-like developed in about 10% of transgenic *E2A-HLF/Mb1*.*Cre* and was characterized by lymphadenopathy starting at six months of age (S4A Fig). Complete blood counts from moribund mice revealed leukocytosis and anemia (Fig 5B), and necropsies showed splenomegaly and hepatomegaly (Fig 5C, S4B Fig). Peripheral blood smears and cytospins from spleen and bone marrow showed mainly mature myeloid cells (Fig 5D). Infiltrating mature myeloid cells were found in several tissues including bone marrow, spleen, liver, lymph nodes, kidney and thymus (S4C Fig). Total bone marrow cells were increased compared to healthy *E2A-HLF/Mb1*.*Cre* transgenic mice and similar to wild-type mice (S5A Fig). Immunophenotypic profile confirmed co-expression of Gr1 and Mac1 antigens in a homogenenous bone marrow cell population (S5B and S5C Fig). To assess the proliferative capacity of the abnormal cells, bone marrow was re-plated in methylcellulose containing myeloid cytokines. Although colonies were formed after the first and second rounds, cells were not able to form colonies after the third round (Fig 5E). Secondary transplantation into sublethally irradiated hosts showed that bone marrow cells from sick mice were unable to reconstitute the disease or to engraft after a 10-month follow-up, consistent with the mature phenotype of malignant cells (Fig 5F). As controls for successful bone marrow transplantation, we performed secondary bone marrow transplantations of bone marrow cells from *E2A-PBX1* transgenic mice [30], which displayed a 100% penetrance for secondary leukemias. Thus, *E2A-HLF/Mb1*.*Cre* mice have a reduced overall survival due to a myeloproliferative-like disease (MPD-like) with low penetrance.

## Discussion

To investigate the in vivo role and molecular pathogenesis of *E2A-HLF*, we developed conditional knock-in mice, in which E2A-HLF expression is directed to the B-cell compartment by Cre-mediated recombination using the *CD19* and *Mb1* promoters and to the HSC compartment by the *Mx1* promoter. Conditional E2A-HLF expression induced B-cell lymphopenia and hyposplenia, due to enhanced B-cell progenitor death, whereas conditional E2A-HLF expression in HSCs was embryonic lethal.

The phenotypes observed in the conditional *E2A-HLF* mouse models are similar to previously reported transgenic mice by our group and others in which E2A-HLF expression was driven by an Ig enhancer and promoter [20] or Eμ enhancer and SV40 promoter [19], which induced apoptosis and cell death in expressing cells and lymphoid hypoplasia. The induction of cell death by E2A-HLF suggests that E2A-HLF must cooperate with pro-survival genes to induce leukemia. Several candidate *E2A-HLF* cooperating genes have been implicated in B-ALL using retroviral insertional mutagenesis in a conditional mouse model [21]. We hypothesize that cooperating genetic events occur prior to or very rapidly after the acquisition of the t(17;19) translocation in B-cell progenitors. Another possibility is that the translocation originates in a “permissive” progenitor B cell, which has not been targeted in the *CD19*.*Cr*e and *Mb1*.*Cre* knock-in mice. The induction of cell death by E2A-HLF in transgenic mice contrasts with the E2A-HLF anti-apoptotic effects observed in several in vitro experimental systems [31–34] raising the possibility that the effects of E2A-HLF on cell survival pathways are context dependent. Although we observed a modest increase of apoptosis after in vitro culture, additional mechanisms causing cell death in conditional E2A-HLF transgenic mice require further investigation.

It has been postulated that *E2A-HLF* oncogenic properties might be due to the dual effects of *E2A* loss of function in conjunction with induction of target genes by the fusion protein [18]. Therefore, we investigated in sorted B-cell progenitors from transgenic mice the expression of known *E2A-HLF* target genes including *Nfil3* [35], *Slug* [36], *Lmo2* [37,38], *Bcl2* [38,39], *Birc5* [40], cell death receptor *Dr5* [41] and *Zfp521* [21]. Increased expression levels were only detected for *Birc5* (p-value<0.05) and *Lmo2* (p-value< 0.01) in E2A-HLF-expressing versus wild type B-cell progenitors (S6 Fig). The increased expression of these genes was not due to E2A haplo-insufficiency since similar differences in their expression levels were not observed in E2A-PBX1 conditional mice using a similar strategy [30]. E2A-HLF protein levels are much higher in B-cell progenitors of transgenic mice compared to an E2A-HLF+ human cell line (Fig 1C), which contrasts to similar transcriptional expression levels compared to *E2A* (S6 Fig). We speculate that E2A-HLF protein is more stable in B-cell progenitors of transgenic mice and thus might interfere with physiological gene regulation of other candidate E2A-HLF target genes in B-cell progenitors, explaining the phenotypic features of the conditional E2A-HLF knock-in mice.

Abnormal lymphocyte development has been observed previously in *E2A-Pbx1* [42] and *E2A-Hlf* transgenic mice [20] and has been attributed to dominant-negative effects of the fusion proteins causing *E2A* loss-of-function, as shown in *E2A*-deficient mice [10,11]. Furthermore, it has been described that down-regulation of *E47* consistent with increased apoptosis and cell death [43]. Although our conditional *E2A-HLF* mouse models show decreased numbers of mature and progenitor B cells likely due to enhanced cell death, a similar conditional mouse model expressing *E2A-PBX1* [30] showed decreased numbers of mature B cells but without displaying increased cell death (data not shown). These data suggest a specific effect of the chimeric fusion proteins on lymphocyte development, which might not only be due to *E2A* haploinsufficiency. However, transcriptional regulation of E2A-HLF target genes and induction of cell death in B cell progenitors of conditional E2A knock-out mice are open questions that should be addressed in future studies.

T cell leukemias were not induced in contrast to previously reported *E2A-HLF* transgenic mice [19,20] however an MPD-like disorder developed in about 10% of *E2A-HLF/Mb1*.*Cre* knock-in mice. We detected GFP+ myeloid precursors in bone marrow (20%) of healthy *E2A-HLF/Mb1*.*Cre* knock-in mice, likely due to leakiness of the Cre-driver promoter, which might explain the oncogenic transformation mediated by E2A-HLF in myeloid cells. Indeed, E2A-HLF is a potent oncogene in myeloid transformation assays using retroviral vectors [44].

In summary, we have developed a conditional *E2A-HLF* mouse model, which can be used as a platform in future studies to validate the in vivo role of previously described cooperating partners (e.g. Bcl2) or novel *E2A-HLF* cooperating genes to further elucidate E2A-HLF leukemogenesis and translational biology.

## Supporting Information

S1 Fig E2A-HLF conditional mice show GFP expression in immature myeloid Gr1+Mac1- cells. Dot plots show gating strategy to analyze GFP expression in myeloid compartment of bone marrow from two transgenic E2A-HLF/Mb1.Cre mice.
(TIFF)

S2 Fig Conditional E2A-HLF transgenic mice develop B cell lymphopenia and hyposplenia. **(A)** Total spleen cells from 3-month-old wild-type (WT, n = 3) and E2A-HLF/Mb1.Cre (n = 3) transgenic mice were enumerated by trypan blue exclusion assay. Horizontal bars denote the mean. Statistical analysis was done by Mann-Whitney U test. **(B)** Dot plots from flow cytometry analysis show mature B cell (B220+CD19+) subpopulations and progenitor B cell subpopulations (Lin-CD19-CD43+, Lin-CD19+CD43+, Lin-CD19+CD43-) in spleen of representative wild-type and E2A-HLF/Mb1.Cre transgenic mice. Lin, lineage markers (CD3, CD4, CD8, NK1-1, Mac1, Gr1, Ter119). **(C)** Graph summarizes relative frequencies of mature B cells and progenitor B cells in spleen of 3-month-old wild-type (n = 3) abd E2A-HLF/Mb1.Cre transgenic mice (n = 3). Columns denote mean and bars denote standard error of the mean.
(TIFF)

S3 Fig E2A-HLF conditional mice show increased B cell progenitor death. **(A)** Dot plots show gating strategy for annexin V (AnnV) and propidium iodide (PI) staining in CD19+CD43+ gated cells of representative wild type, E2A-HLF/Mb1.Cre 2-month-old and 6-month-old transgenic mice.
(TIF)

S4 Fig E2A-HLF/Mb1.Cre transgenic mice develop MPD-like disorder. **(A, B)** Images show cervical lymphadenopathy **(A)** and spleen enlargement **(B)** from representative MPD-like mouse. **(C)** Histologic analysis after hematoxylin-eosin staining shows infiltrating cells in the indicated tissues.
(TIFF)

S5 Fig Increased myeloid progenitor subpopulations in E2A-HLF/Mb1.Cre mice. **(A)** Total bone marrow cells from healthy (n = 11) and MPD-like (n = 6) conditional E2A-HLF/Mb1.Cre mice were enumerated by trypan blue exclusion assay. Horizontal bars denote the mean. Statistical analysis was done by Mann-Whitney U test. **(B)** Flow cytometry analysis of bone marrow from representative wild type (WT) and myeloproliferative disease like (MPD-like) mice, shows forward and side scatter (left panel) and myeloid markers Gr1 and Mac1 (right panel). **(C)** Myeloid cell progenitors from bone marrow (BM) of wild type (n = 8), healthy E2A-HLF/Mb1.Cre (n = 3), and E2A-HLF/Mb1.Cre MPD-like mice (n = 3) were analyzed by flow cytometry using Gr1 and Mac1 conjugated antibodies. Statistical analysis was performed by student’s t-test, ** p-value <0.01, * p-value <0.05 and n.s, not significant.
(TIFF)

S6 Fig Transcriptional analysis of E2A-HLF target genes in B cell progenitors. **(A)** B cell progenitors (Lin-CD19+CD43+) were FACS-sorted from bone marrow of wild–type (n = 3), E2A-HLF transgenic (GFP+, n = 3) and E2A-PBX1 transgenic (GFP+, n = 3) mice. Expression of control (*E2A*, *E2A-HLF* and *HLF*), and E2A-HLF target genes (*Birc5*, *Lmo2*, *Bcl2*, *Zfp521*, *Dr5*, *Nfil3* and *Slug*) was analyzed by RTqPCR using ΔΔCt method. *Actb* was used as housekeeping gene. Statistical analysis was performed by Mann-Whitney U test between wild type and E2A-HLF/Mb1.cre transgenic mice. * denotes a p-value <0.01, ** p-value<0.05.
(TIFF)

S1 Table Antibodies for flow cytometry analysis and FACS. (DOCX)

S2 Table Primers for RT qPCR. (DOCX)
