# Supplementary figures and images for: Conditional Expression of E2A-HLF Induces B-Cell Precursor Death and Myeloproliferative-Like Disease in Knock-In Mice
Source: PLoS One. 2015 Nov 20;10(11):e0143216. doi: 10.1371/journal.pone.0143216 (PMC4654581; doi:10.1371/journal.pone.0143216)

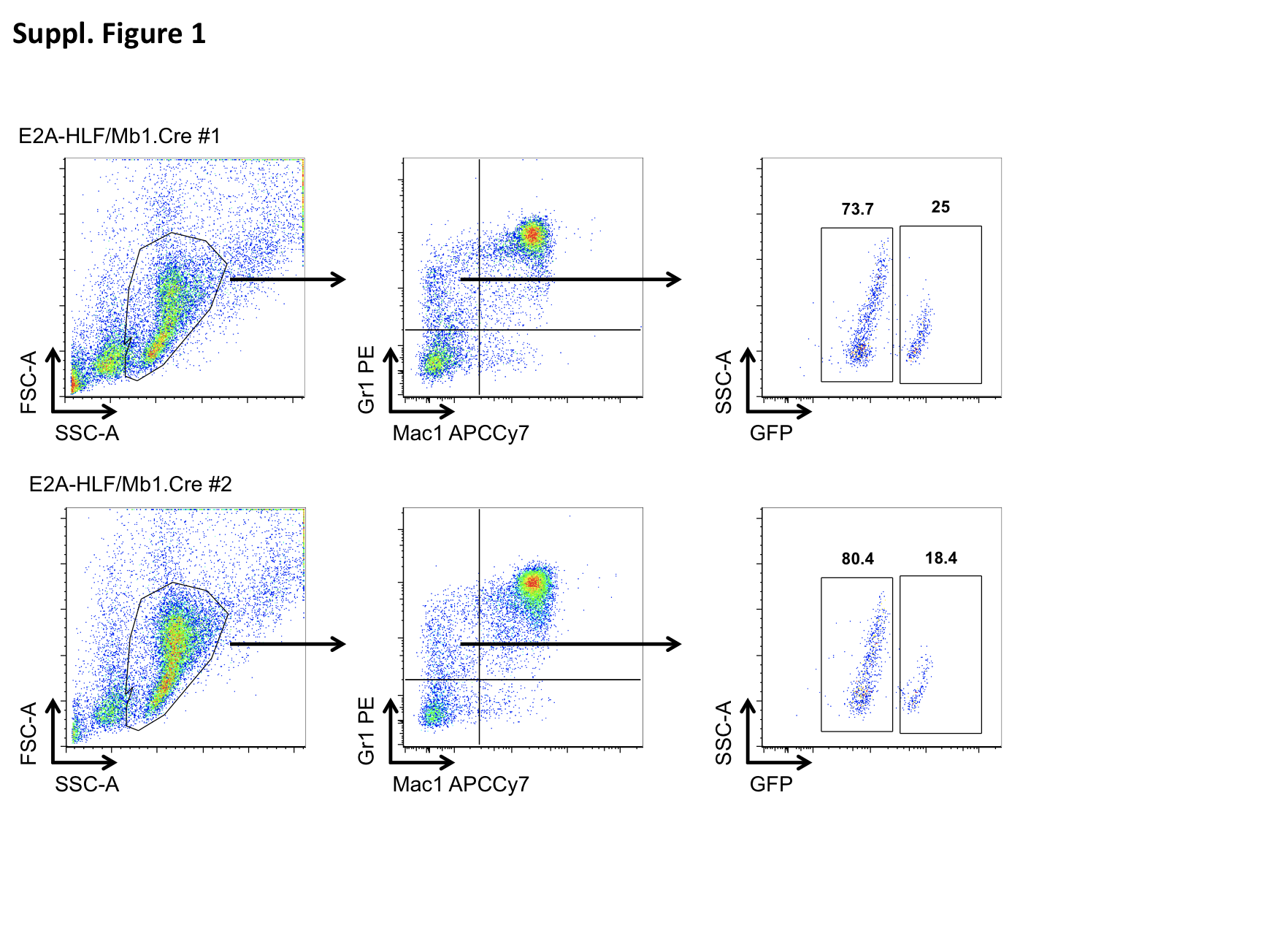

Supplement: S1 Fig — Dot plots show gating strategy to analyze GFP expression in myeloid compartment of bone marrow from two transgenic E2A-HLF/Mb1.Cre mice. (TIFF) [file pone.0143216.s001.tiff]

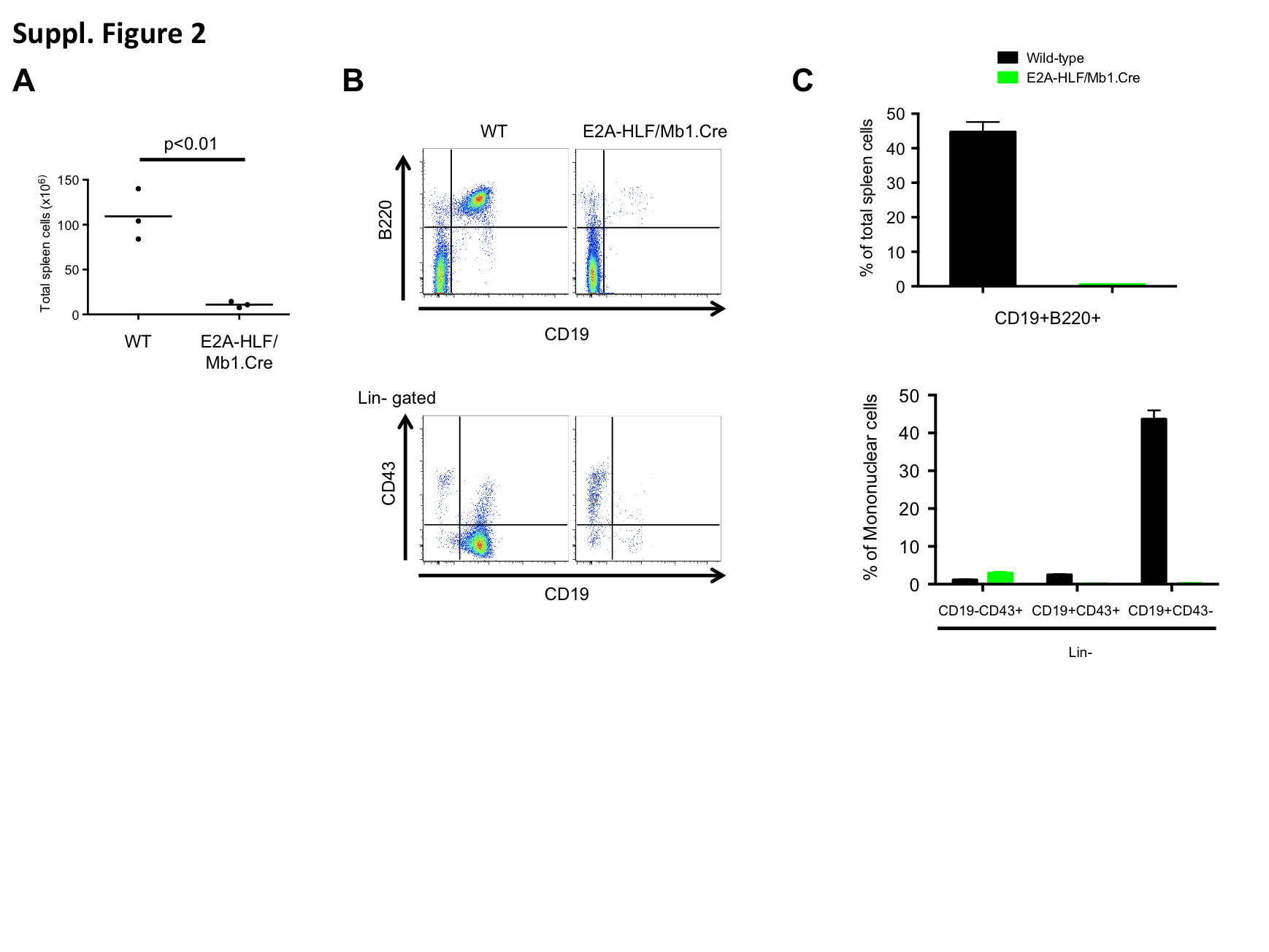

Supplement: S2 Fig — (A) Total spleen cells from 3-month-old wild-type (WT, n = 3) and E2A-HLF/Mb1.Cre (n = 3) transgenic mice were enumerated by trypan blue exclusion assay. Horizontal bars denote the mean. Statistical analysis was done by Mann-Whitney U test. (B) Dot plots from flow cytometry analysis show mature B cell (B220+CD19+) subpopulations and progenitor B cell subpopulations (Lin-CD19-CD43+, Lin-CD19+CD43+, Lin-CD19+CD43-) in spleen of representative wild-type and E2A-HLF/Mb1.Cre transgenic mice. Lin, lineage markers (CD3, CD4, CD8, NK1-1, Mac1, Gr1, Ter119). (C) Graph summarizes relative frequencies of mature B cells and progenitor B cells in spleen of 3-month-old wild-type (n = 3) abd E2A-HLF/Mb1.Cre transgenic mice (n = 3). Columns denote mean and bars denote standard error of the mean. (TIFF) [file pone.0143216.s002.tiff]

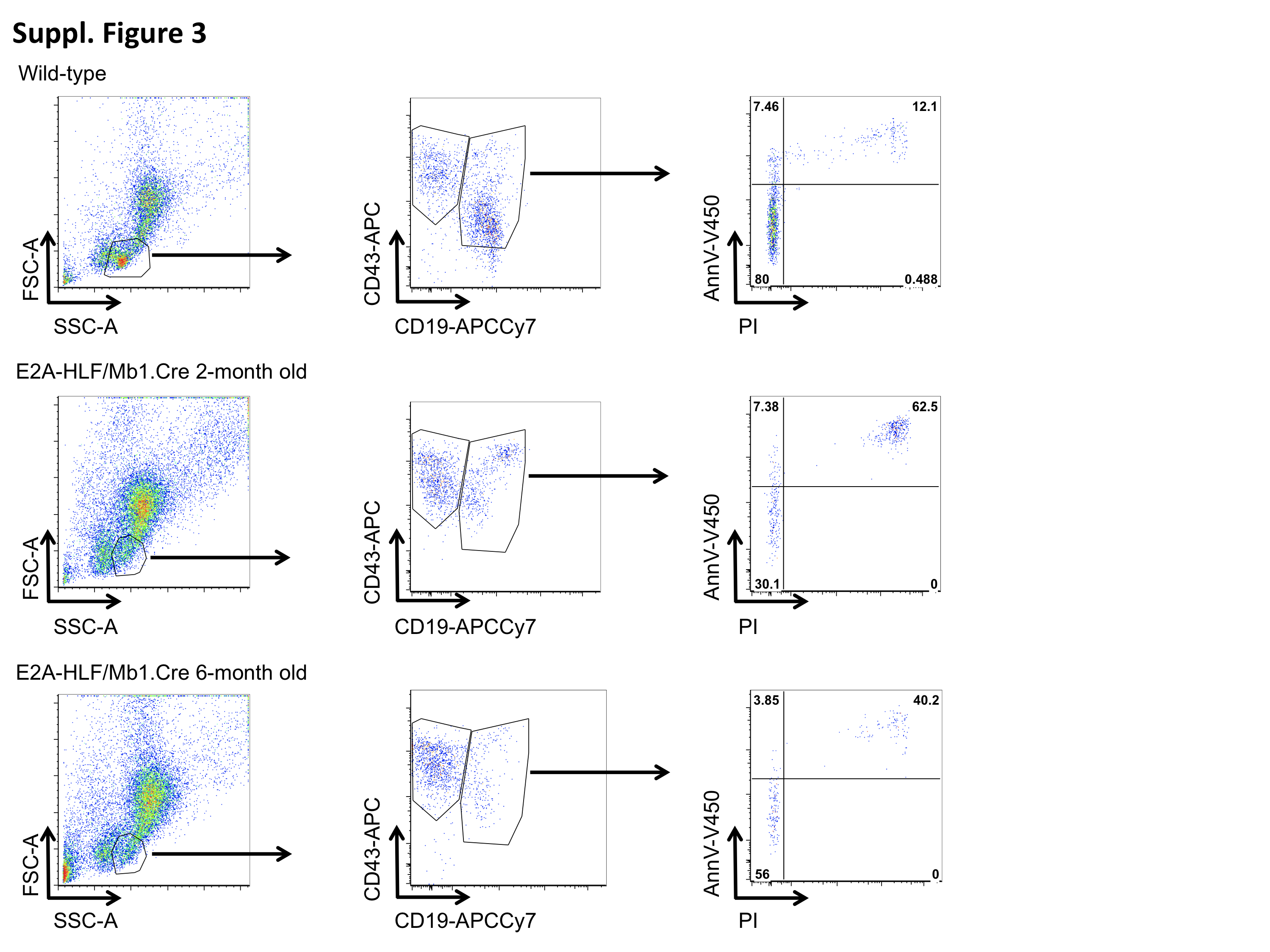

Supplement: S3 Fig — (A) Dot plots show gating strategy for annexin V (AnnV) and propidium iodide (PI) staining in CD19+CD43+ gated cells of representative wild type, E2A-HLF/Mb1.Cre 2-month-old and 6-month-old transgenic mice. (TIF) [file pone.0143216.s003.tif]

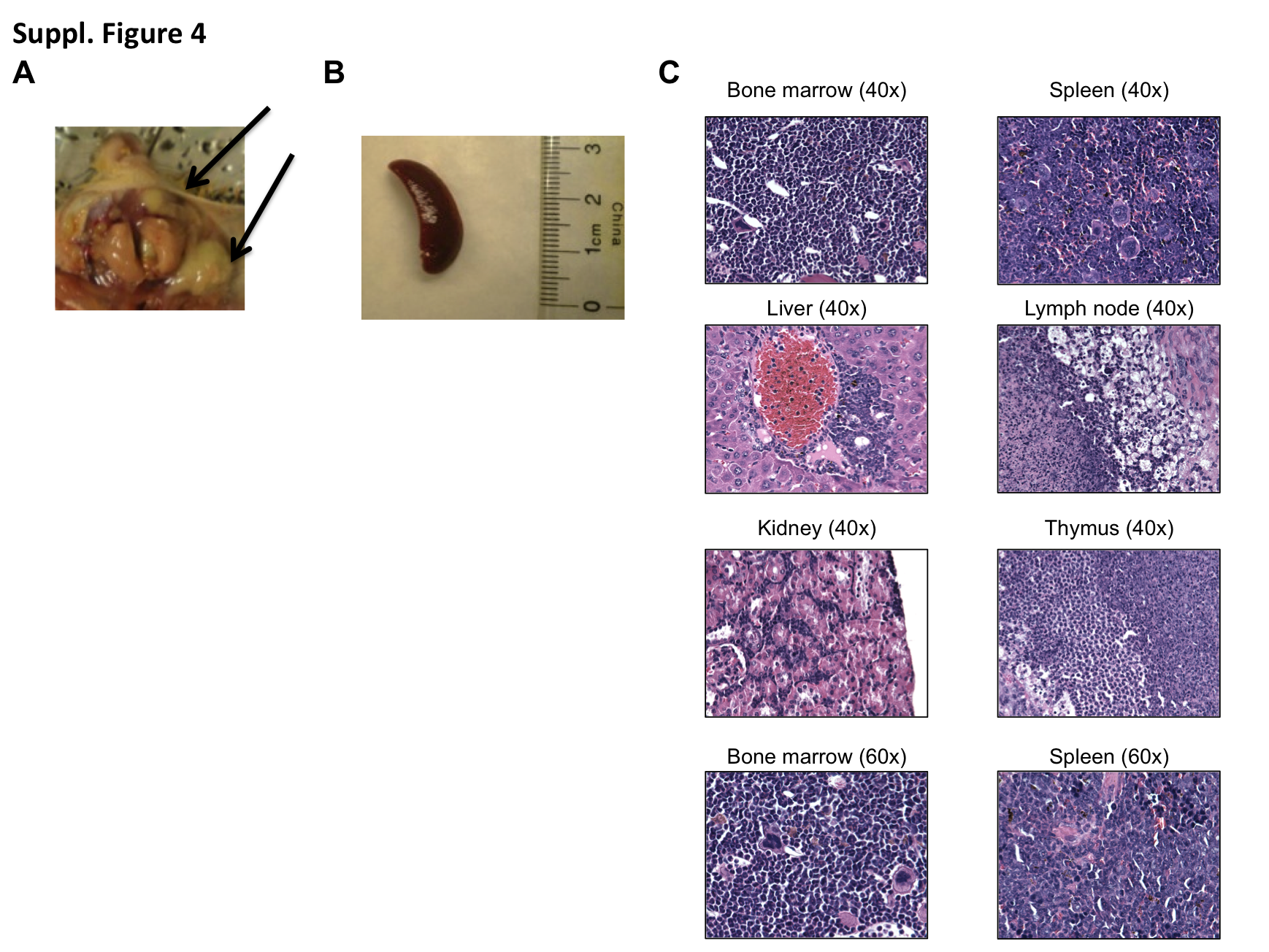

Supplement: S4 Fig — (A, B) Images show cervical lymphadenopathy (A) and spleen enlargement (B) from representative MPD-like mouse. (C) Histologic analysis after hematoxylin-eosin staining shows infiltrating cells in the indicated tissues. (TIFF) [file pone.0143216.s004.tiff]

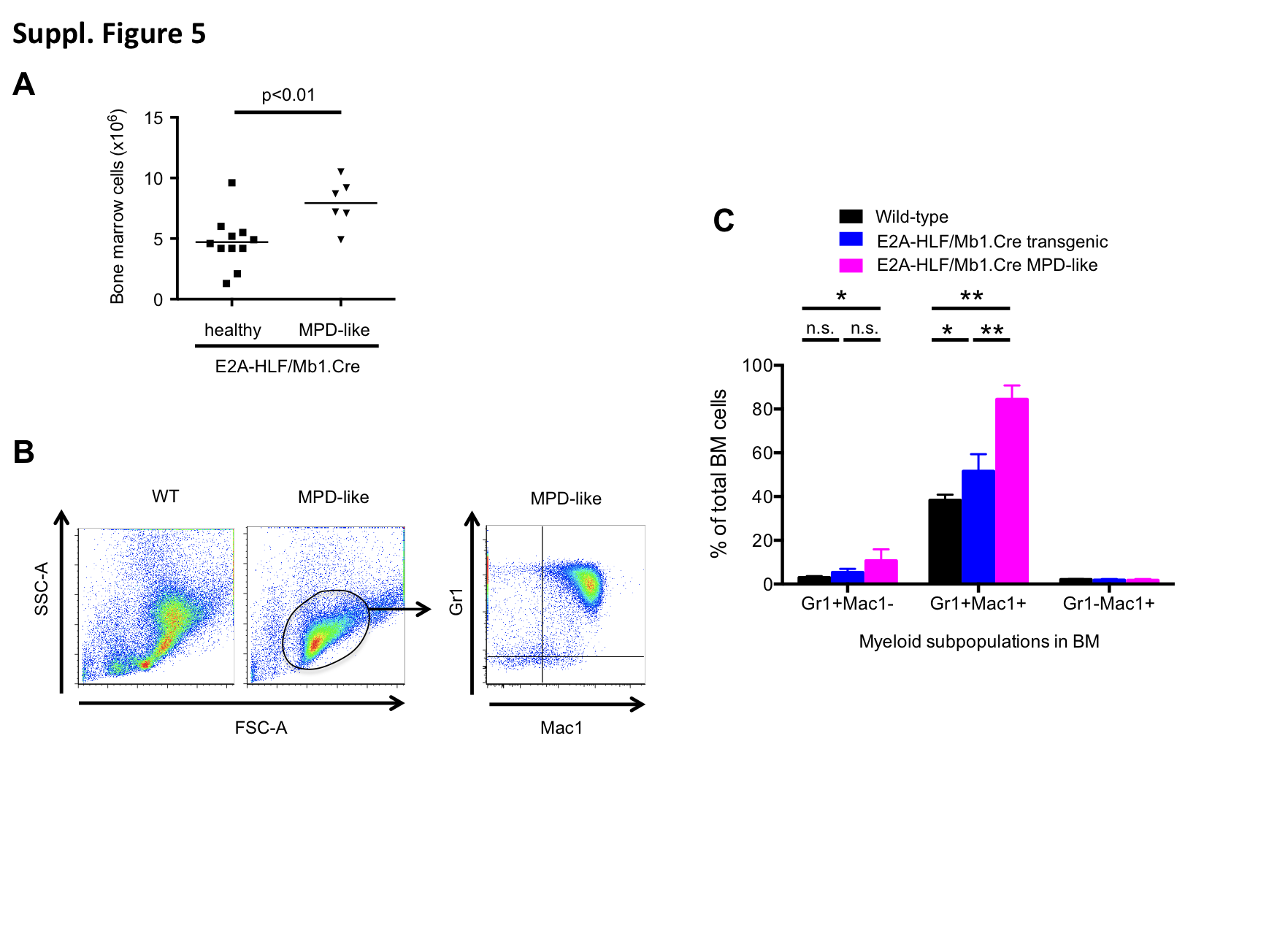

Supplement: S5 Fig — (A) Total bone marrow cells from healthy (n = 11) and MPD-like (n = 6) conditional E2A-HLF/Mb1.Cre mice were enumerated by trypan blue exclusion assay. Horizontal bars denote the mean. Statistical analysis was done by Mann-Whitney U test. (B) Flow cytometry analysis of bone marrow from representative wild type (WT) and myeloproliferative disease like (MPD-like) mice, shows forward and side scatter (left panel) and myeloid markers Gr1 and Mac1 (right panel). (C) Myeloid cell progenitors from bone marrow (BM) of wild type (n = 8), healthy E2A-HLF/Mb1.Cre (n = 3), and E2A-HLF/Mb1.Cre MPD-like mice (n = 3) were analyzed by flow cytometry using Gr1 and Mac1 conjugated antibodies. Statistical analysis was performed by student’s t-test, ** p-value <0.01, * p-value <0.05 and n.s, not significant. (TIFF) [file pone.0143216.s005.tiff]

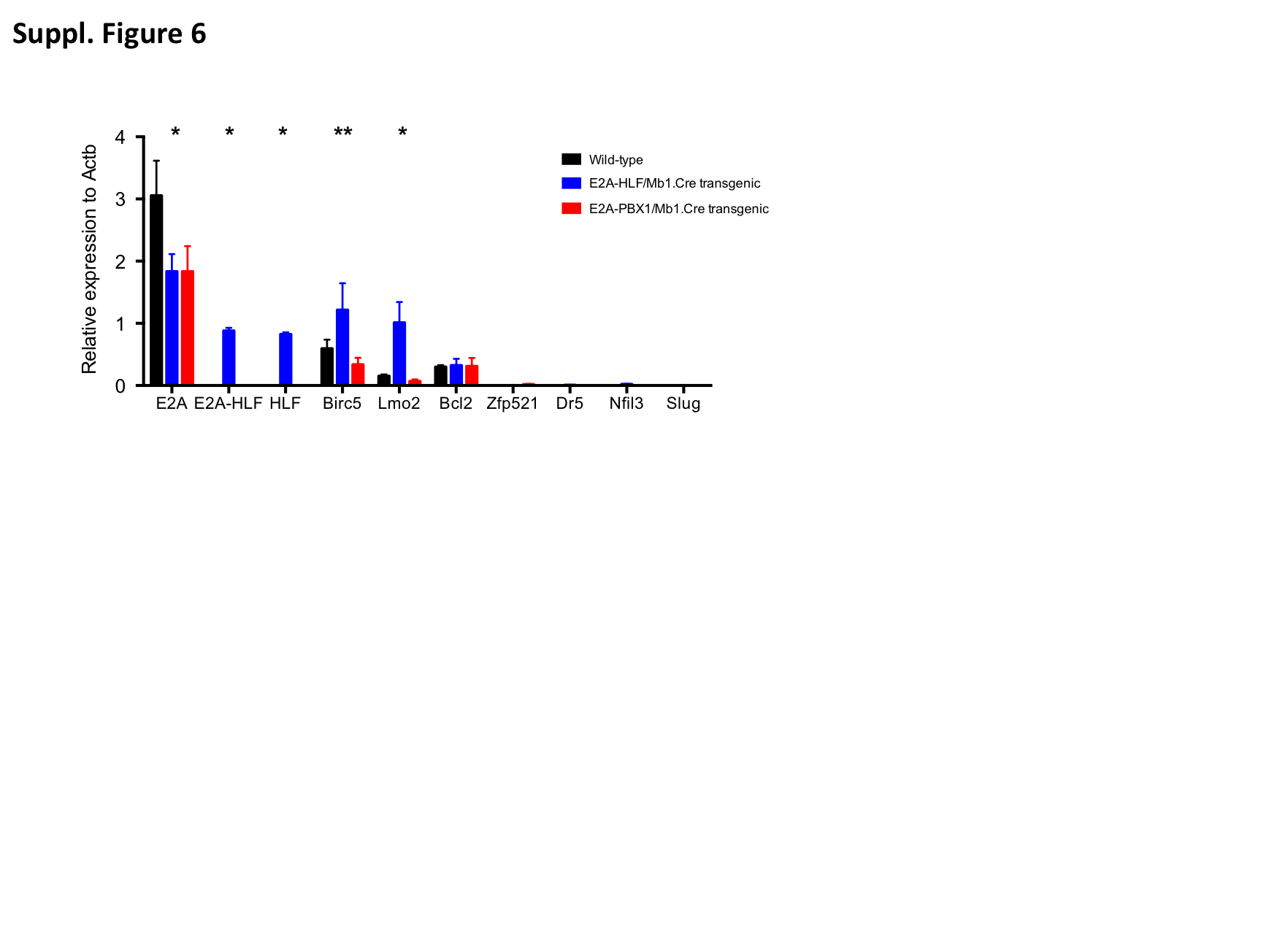

Supplement: S6 Fig — (A) B cell progenitors (Lin-CD19+CD43+) were FACS-sorted from bone marrow of wild–type (n = 3), E2A-HLF transgenic (GFP+, n = 3) and E2A-PBX1 transgenic (GFP+, n = 3) mice. Expression of control (E2A, E2A-HLF and HLF), and E2A-HLF target genes (Birc5, Lmo2, Bcl2, Zfp521, Dr5, Nfil3 and Slug) was analyzed by RTqPCR using ΔΔCt method. Actb was used as housekeeping gene. Statistical analysis was performed by Mann-Whitney U test between wild type and E2A-HLF/Mb1.cre transgenic mice. * denotes a p-value <0.01, ** p-value<0.05. (TIFF) [file pone.0143216.s006.tiff]
