# Supplementary material for: Conditional Expression of E2A-HLF Induces B-Cell Precursor Death and Myeloproliferative-Like Disease in Knock-In Mice
Source: PLoS One. 2015 Nov 20;10(11):e0143216. doi: 10.1371/journal.pone.0143216 (PMC4654581; doi:10.1371/journal.pone.0143216)
Supplement: S1 Table — (DOCX) [file pone.0143216.s007.docx]

| **Supplementary Table 1. Antibodies for flow cytometry analysis and FACS.** | | | |
| --- | --- | --- | --- |
| **Antigen** | **Fluorochrome** | **Clone** | **Source** |
| CD19 | APC-Cy7 | 1D3 | BD Biosciences |
| CD43 | APC | S7 | BD Biosciences |
| CD43 | PE | S7 | BD Biosciences |
| CD3 | PE | 145-2C11 | BD Biosciences |
| CD4 | PE | RM4-5 | BD Biosciences |
| CD8 | PE | E53-6.7 | eBiosciences |
| Mac1/CD11b | PE | M1/70 | BD Biosciences |
| Gr1 | PE | RB6-8C5 | BD Biosciences |
| NK1.1 | PE | PK136 | eBiosciences |
| Ter119 | PE | TER-119 | eBiosciences |
| CD45R/B220 | PECy7 | RA3-6B2 | BD Biosciences |
| Anti-cleaved Caspase 3 | PE | C92-605 | BD Pharmingen |
| Mac1/CD11b | APCCy7 | M1/70 | BD Biosciences |
