# Supplementary material for: Conditional Expression of E2A-HLF Induces B-Cell Precursor Death and Myeloproliferative-Like Disease in Knock-In Mice
Source: PLoS One. 2015 Nov 20;10(11):e0143216. doi: 10.1371/journal.pone.0143216 (PMC4654581; doi:10.1371/journal.pone.0143216)
Supplement: S2 Table — (DOCX) [file pone.0143216.s008.docx]

| **Supplementary Table 2. Primers for RT qPCR.** | | |
| --- | --- | --- |
| **Gene name** |  | **Sequence** |
| *Actb* | FW | CGTGAAAAGATGACCCAGATC |
|  | RW | CACAGCCTGGATGGCTACGT |
| *E2A* | FW | ATGCCCTTGGGAAGGCACTG |
|  | RW | CCATGGAGACCTGCATCGTAG |
| *E2A-HLF* | FW | CCCTCTCCACCTCGATCTACT |
|  | RW | ATGTTGTTCTTTCTGCGCCT |
| *HLF* | FW | CGGAGGCTGAAAGAGAACCA |
|  | RW | CCCAGCTCCTTCCTCAAGTC |
| *Survivin/Birc5* | FW | CTTCATCCACTGCCCTACCG |
|  | RW | GTTGGTCTCCTTTGCAATTTTGT |
| *Lmo2* | FW | CTGTGACCTCTGTGGGTGTC |
|  | RW | GCACAGAGACCATCCTGACC |
| *Bcl2* | FW | GTCGCTACCGTCGTGACTTC |
|  | RW | CAGACATGCACCTACCCAGC |
| *Zfp521/Evi3* | FW | CGAGTCCCTGCGGGTTAG |
|  | RW | GGGTCTTTGAGGGATCTCGG |
| *Dr5* | FW | AAAACGGCTTGGGCATCTTG |
|  | RW | TGTCGGTCCAAGAGAGACGA |
| *Nfil3* | FW | CAGCCGCCCTTTCTTTTCCC |
|  | RW | AATGGGTCCTTCTGTTGTCCG |
| *Slug/Snai2* | FW | TGGCTGCTTCAAGGACACATT |
|  | RW | GTGCCCTCAGGTTTGATCTG |
